# Computed tomography-guided permanent brachytherapy for locoregional recurrent gastric cancer

**DOI:** 10.1186/1748-717X-7-114

**Published:** 2012-07-24

**Authors:** Liangrong Shi, Changping Wu, Jun Wu, Wenjie Zhou, Mei Ji, Hongyu Zhang, Jiemin Zhao, Yuanquan Huang, Honglei Pei, Zhong Li, Jingfang Ju, Jingting Jiang

**Affiliations:** 1 Department of Oncology, The Third Affiliated Hospital, Soochow University, 185 Juqian Street, Changzhou 213003, Jiangsu Province, China; 2 Department of Radiology, the Third Affiliated Hospital, Soochow University, Changzhou 213003, Jiangsu Province, China; 3 Department of Radiation Oncology, the Third Affiliated Hospital, Soochow University, Changzhou 213003, Jiangsu Province, China; 4 Department of Gastrointestinal Surgery, the Third Affiliated Hospital, Soochow University, Changzhou, 213003, Jiangsu Province, China; 5 Department of Pathology, Stony Brook University, Stony Brook, NY, 11794, USA

**Keywords:** Gastric cancer, Surgery, Locoregional recurrence, Brachytherapy, Iodine-125 seed

## Abstract

**Background:**

Locoregional recurrence is the typical pattern of recurrence in gastric cancer, and cannot be removed by surgery in most of the patients. We aimed to evaluate the feasibility and efficacy of computed tomography (CT)-guided brachytherapy for patients with locoregional recurrent gastric cancer.

**Materials and methods:**

We reviewed the case histories of 28 patients with locoregional recurrent gastric cancer that were selected for CT- guided brachytherapy by a multidisciplinary team. The clinical data of the patients including patient characteristics, treatment parameters, short-term effects, and survival data were collected and analyzed.

**Results:**

15-75 ^125^I seeds were implanted into each patient to produce a minimal peripheral dose (MPD) 100-160 Gy. Median day 0 dosimetry was significant for the following: V100 (the volume treated with the prescription dose) 95.8% (90.2-120.5%) and D90 (prescription dose received by at least 90% of the volume) 105.2% (98.0-124.6%) of prescription dose. No serious complications occurred during the study. Two months after brachytherapy, complete response, partial response and progressive disease were observed in 50.0%, 28.6% and 21.4% of patients, respectively. The median survival time was 22.0 ± 5.2 months, and the 1, 2,and 3-year survival rate was 89 ± 6%, 52 ± 10% and 11 ± 7%, respectively. A univariate analysis showed that the tumor size was a significant predictor of overall survival (*P* = 0.034). Patients with tumors <3 cm had relatively higher complete response rate (66.7%), compared to those with tumors >3 cm (30.8%). The PTV (planning target volume) smaller than 45 cm^3^ was significantly correlated with achieving complete tumor eradication in the treated region (*P* = 0.020).

**Conclusions:**

For selected patients with limited locoregional recurrent gastric cancer, CT-guided brachytherapy using ^125^I seeds implantation can provide a high local control rate, with minimal trauma.

## Introduction

Despite a remarkable decline of the incidence during the second half of the 20th century, gastric cancer still remains as the fourth most common cancer worldwide and the second most common cause of cancer-related death [1]. Nearly two-thirds of gastric cancer cases occur in developing countries and 42% in China alone [2]. The prognosis of locally advanced gastric cancer remains dismal even after potentially curative resection, adjuvant chemotherapy and radiotherapy [3]. The 5-year overall survival (OS) rates remain between 30% to 40% [3,4]. Tumor recurrence is the main cause of the failure of treatment. Prospective studies have shown that the recurrence of gastric cancer was most commonly involved with locoregional recurrence, heamatogenous metastasis, and peritoneal lesions [5-7]. Most of the patients with recurrent gastric cancer present advanced progression, such as multiple heamatogenous metastasis, extensive lymphatic. In such cases, systemic chemotherapy is the only potential treatment available to patients. However, for the patients with solitary locoregional recurrence, a survival benefit may be expected from local treatment such as surgery [8,9], and external beam radiotherapy (EBRT) [10,11], as opposed to systemic chemotherapy alone.

Brachytherapy (permanent implantation of radioactive seed) has emerged as an alternative local treatment for solid tumors, and is widely applied for its curative effects, minimal trauma, and few associated complications [12-17]. In this study, we aimed to evaluate the feasibility and efficacy of computed tomography (CT)-guided brachytherapy for selected patients with locoregional recurrent gastric cancer. Our group included those with: solitary recurrence at the primary tumor bed, single regional lymph node recurrence and solitary intra-abdominal peritoneal recurrence.

## Materials and methods

### Patients

Patient inclusion criteria include: 1). Gastric cancer patients with limited locoregional recurrence including solitary recurrence at primary tumor bed, solitary intra-abdominal peritoneal recurrence, and single regional lymph node recurrence (no more than 3 lymph nodes) based on diagnosis by CT, and confirmed by percutaneous puncture biopsy; 2). A performance score of no more than 2 according to the criteria of the Eastern Cooperative Oncology Group (ECOG); 3). Absence of intraluminal recurrence determined by gastroscope; 4). No previous history of abdominal radiotherapy. Whether CT-guided brachytherapy was indicated in a patient was determined by the Gastric Cancer Collaborative Group (GCCG) of the hospital, which was composed of a medical oncologist, radiologist, surgeon, and intervention specialist. The multidisciplinary assessment included the potential value of brachytherapy in improving local control and survival, the risks of seed implantation, the identification of vital structures surrounding the recurrent tumor, and the accessibility of safe puncture ways to ensure an optimal dose distribution in the treatment area.

All the patients provided informed consent, which was approved by the Ethics Committee of the Third Affiliated Hospital of Soochow University.

### Instruments and materials

CT scanning machine: 16-spiral CT scanner (Siemens) and Treatment planning system (TPS): HGGR-2000 (Hokai Medical Instrument Co., Ltd, ZhuHai, China), Implantation instruments: 18-G needles (Dr.J, Japan) and implantation gun (JACO Pharmaceutical Co., Ltd, NingBo, China), ^125^I sealed seed sources (JACO Pharmaceutical Co.,Ltd, NingBo, China). The seeds were manufactured from silver rods, which absorbed ^125^I, and were enclosed in a laser- welded titanium capsule. Each seed was 0.8 mm in diameter, 4.5 mm in length and had a capsule wall thickness of 0.05 mm. The ^125^I produces gamma rays (24-35 keV) with a half-life of 59.6 days, half-value thickness of 0.025 mm of lead, penetration of 17 mm, incipient rate of 7 cGy/h, and activities of 0.5-0.8 mCi. All ^125^I seeds were mailed to our hospital in a type-A pack that passed through leak detection and activity tests.

### CT-guided implantation protocol

The three-dimensional images of the tumor were reconstructed with the TPS based on data from CT images. PTV (planning target volume) was defined as GTV (gross tumor volume) plus 0.5-1.5 cm in each direction. PTV was covered by 90% of isodose curves. This dose was prescribed as the minimal peripheral dose (MPD) encompassing the PTV. The total seeds strength that needed to be implanted was calculated by the TPS. Seeds were implanted at least 1.0 cm away from the intestinal wall, and the maximum 2 cm^3^ dose to small intestine was lower than 60 Gy. In practice, 15% more seeds were implanted than planned to ensure the maximum radiation effect.

Patients fasted for 24 h prior to the operation, and oral laxatives were given 12 h before the procedure. Patients received intramuscular injection of diazepam (10 mg) and anisodamine (10 mg) 30 minutes before the operation. All the brachytherapy implants were performed in a standard CT room. Before the implantation procedure, the patient underwent scanning at reconstruction intervals of 5 mm and dual-phase enhanced-contrast. A safe puncture path was carefully determined according to the CT images. Our preferred puncture paths were natural gaps or spaces such as the paraspinal or perirenal spaces. In addition, in cases where tumors were located in the porta hepatic or near the right wall of abdominal aorta, we penetrated the liver. For tumors in the omentalis or the head of the pancreas we traversed the stomach; and for tumors in the hilum of the spleen on in the tail of the pancreas, we traversed the colon. The puncture points on the skin were marked according to CT images. The abdominal wall was anesthetized with 2% lidocaine. For the puncture procedure, the patient was instructed to hold his/her breath at the end of a normal expiration. The needle was placed to a depth determined at each point by the CT scan. Repeated CT scanning at intervals of 5 mm with needle in place permitted the adjustment of depth and angle of needle direction, and avoided puncturing vessels, pancreatic duct and bile duct. Adjacent needles were separated by about 1.0-1.5 cm. The ^125^I seeds were inserted through each needle by using the implantation gun. Each needle was withdrawn 0.5-1.0 cm, and another radioactive seed was inserted. Post-placement CT was performed to document the distribution of seeds in the tumor immediately after seed implantation. The practical radiation dose was validated by the TPS. The dose-volume histogram (DVH) parameters including D90 (prescription dose received by at least 90% of the volume) and V100 (the volume treated with the prescription dose) were recorded. In case of an unsatisfactory dose distribution, the optimization parameters were adjusted and the calculation was repeated using the TPS. Additional ^125^I seed implant will be performed immediately if puncturing the low-dose region in PTV was feasible.

### Continuous treatment after seeds implantation

When a residual tumor or a new solitary locoregional recurrence was detected after brachytherapy, repeated seed implantation might be performed in the patient after reassessment. Although most patients with recurrent gastric cancer have a prior history of chemotherapy after surgery, fluoropyrimidine-based chemotherapy was routinely recommended after brachytherapy.

### Evaluation of the short-term effects and follow up

Assessment for tumor response was carried out 2 months after the ^125^I seeds implantation. Complete response (CR, no residual tumor in the area with radioactive seeds completely gathering together), partial response (PR), stable disease (SD) and progressive disease (PD) were reported according to the Response Evaluation Criteria in Solid Tumors (RECIST). The overall response rate (ORR) was the sum of CR and PR.

Follow-up was performed every 3 months after ^125^I seeds implantation. Follow-up consisted of physical examination, spiral computed tomography or ultrasonography, chest radiography, serum biochemistry, and clinical examination. Gastroscopy was also performed for patients in whom intraluminal recurrence was suspected. The median follow-up period was 22 months, ranging from 7 to 39 months.

### Statistical analysis

Overall survival (OS) was defined as the time from the date of brachytherapy to the date of death from any cause or the date of the last follow-up. Participants who were alive at the end of the study period were considered censored. Progression-free survival (PFS) was defined as the time-period from the date of operation to the date of the first tumor progression or the date of last follow-up. Participants who were recurrence-free were considered censored. Continuous data are presented as media ± Standard Deviation. The 1-, 2- and 3-year survival rates were estimated using the Life Table method. The OS curve was estimated using the Kaplan–Meier survival analysis. The following variables were assessed as potential predictors in univariate Cox models of survival: age, gender, time to recurrence, original TNM stage and tumor size. A minimum *P* value approach was used to determine the optimal cut-off points to dichotomize continuous variables of interest, and corresponding *P* values were adjusted using Miller and Siegmund adjustment [18]. Data were analyzed using SPSS software (version 13.0, SPSS Inc., Chicago, IL, USA.).

## Results

### Enrollment

From July 2007 to June 2011,209 patients were diagnosed as having recurrence after gastrectomy for gastric cancer at our hospital. 38 patients (18.1%) presented with limited locoregional recurrence. 3 patients were excluded from seed implantation because the tumor position was not easily accessible to needle puncture (2 patients had tumors located in the gap between the aorta and inferior vena cava and 1 patient had a tumor located in the space between the aorta and the superior mesenteric artery). 2 patients were considered unsuitable for brachytherapy due to poor physical health. The remaining 33 (86.8%) of 38 patients were assessed suitable for seed implantation. Among the 33 patients, 3 patients received EBRT, 2 patients received surgery, and 28 patients eventually received CT-guided brachytherapy.

The study included 18 males and 10 females ranging in age from 42 to 83 years old. Solitary extraluminal recurrence was found in 13 patients, and a single regional lymph node recurrence in 15 patients. The average time to recurrence was 18.0 ± 6.2 months (ranging from 7 to 36 months). 23 patients underwent D2 gastrectomy, and 5 patients underwent D1 gastrectomy. 24 patients received adjuvant chemotherapy after surgery. The details of the patient characteristics are shown in Table 1.

**Table 1:** Patient characteristics

**Case**	**Age/sex**	**Site of recurrence**	**Tumor size (mm)**	**Time to recurrence (month)**	**Previous operation**	**Original stage**	**Original Tumor site**	**LN dissection**	**Original pathology**	**Adjuvant CT**
1	59/M	head of pancreas	30	7	TG	T_4_N_1_M_0_	lower	D2	intestinal	yes
2	65/M	omental bursa	50	11	PG	T_3_N_1_M_0_	lower	D2	intestinal	yes
3	62/F	omental bursa	50	18	PG	T_2_N_2_M_0_	middle	D2	intestinal	yes
4	83/M	porta hepatis	25	32	PG	T_2_N_1_M_0_	lower	D1	intestinal	no
5	45/F	tail of pancreas	60	16	TG	T_4_N_2_M_0_	middle	D2	diffuse	yes
6	66/M	PALN	25	28	PG	T_3_N_2_M_0_	upper	D2	intestinal	yes
7	49/M	PALN	35	17	PG	T_3_N_1_M_0_	lower	D2	intestinal	yes
8	79 M	hilum of spleen	28	10	PG	T_2_N_2_M_0_	lower	D1	intestinal	yes
9	67/F	omental bursa	25	14	TG	T_3_N_1_M_0_	lower	D2	diffuse	yes
10	72/M	omental bursa	45	13	PG	T_2_N_1_M_0_	upper	D2	intestinal	no
11	49/M	head of pancreas	25	14	PG	T_3_N_1_M_0_	lower	D2	intestinal	yes
12	42/M	porta hepatis	40	16	PG	T_3_N_1_M_0_	middle	D2	diffuse	yes
13	66/M	porta hepatis	22	19	PG	T_3_N_1_M_0_	lower	D2	intestinal	yes
14	72/F	head of pancreas	38	10	TG	T_4_N_1_M_0_	lower	D1	diffuse	yes
15	63/M	PALN	20	26	PG	T_2_N_1_M_0_	upper	D2	intestinal	yes
16	70/M	omental bursa	26	18	TG	T_4_N_0_M_0_	lower	D2	diffuse	yes
17	62/F	omental bursa	55	24	PG	T_3_N_1_M_0_	middle	D2	intestinal	yes
18	45/F	tail of pancreas	43	20	PG	T_2_N_2_M_0_	lower	D2	intestinal	yes
19	55/F	PALN	52	16	PG	T_1_N_2_M_0_	upper	D1	intestinal	yes
20	58/M	PALN	22	30	PG	T_3_N_1_M_0_	lower	D2	intestinal	yes
21	71/M	hilum of spleen	45	9	TG	T_$_N_2_M_0_	lower	D2	diffuse	yes
22	62/F	omental bursa	48	24	TG	T_3_N_1_M_0_	middle	D2	intestinal	yes
23	45/M	PALN	25	23	PG	T_2_N_1_M_0_	lower	D2	intestinal	yes
24	68/F	tail of pancreas	38	32	PG	T_2_N_1_M_0_	lower	D2	intestinal	yes
25	71/M	PALN	35	36	PG	T_2_N_1_M_0_	upper	D2	intestinal	no
26	76/M	porta hepatis	22	24	PG	T_4_N_0_M_0_	upper	D1	intestinal	yes
27	49/F	omental bursa	30	11	TG	T_3_N_2_M_0_	middle	D2	diffuse	yes
28	79/M	PALN	20	14	PG	T_3_N_1_M_0_	lower	D1	intestinal	no

PALN:para-aortic lymph node; TG: total gastrectomy; PG: partial gastrectomy; TNM stage: International Union Against Cancer (UICC 2002) TNM system; LN: lymph node; CT: chemotherapy.

### Response and survival

**Figure 1:**
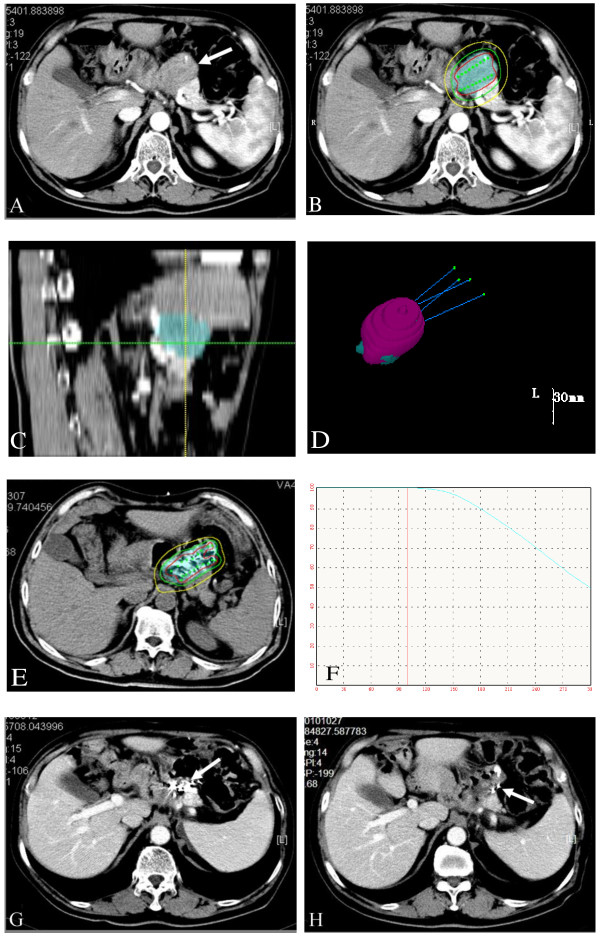
**TPS planning diagram and CT scans during follow-up with recurrence in the primary tumor bed after total gastrectomy. a**). Before treatment, the tumor size was 35 mm in diameter; **b**). Isodose curves for treatment planning (“iso” 120 Gy, red line = 150%; green line =100%; yellow line = 50%); **c**). Sagittal images reconstructed by the TPS based on data from perioperation CT images: the skyblue area represent the PTV; **d**). Three-dimensional view of the TPS planning program: four puncture paths were designed; 99.0% of PTV (the skyblue area) was covered by 90% of isodose curves (the pink area); **e**). A typical CT slice showing the distribution of ^125^I seeds and isodose curves after seed implantation (red line = 180 Gy; green line =120 Gy; yellow line = 60 Gy); **f**). The D0 dose-volume histogram caculated by TPS (V100 = 96.2%, D90 = 117%); **g**). Two months after treatment, the recurrent tumor was completely eradicated, and ^125^I seeds gathered together; **h**). Six months after treatment, there was no progression in the treatment region, and some seeds migrated.

All the target tumors were responsive to the treatment 2 months after ^125^I seeds implantation. The evaluation of the short-term effects was as follows: CR occurred in 14 cases (50.0%), PR in 8 cases (28.6%), and PD in 6 cases with recurrences in new sites (21.4%) (Lymph node metastasis occurred in 3 cases, peritoneal metastasis occurred in 2 cases, and hepatic metastasis occurred in 1 case). ORR for this group of patients was 78.6%. 13 patients (46.4%) had residual tumor in the initial region. One typical patient’s TPS planning progamm and CT scans during follow-up are shown in Figure 1.

**Figure 2:**
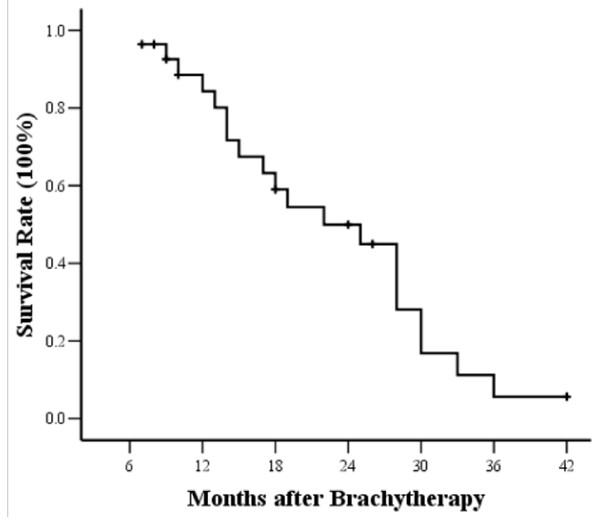
**Kaplan-Meier estimates for overall survival (OS) for patients.** The median survival time was 22.0 ± 5.2 months.

**Figure 3:**
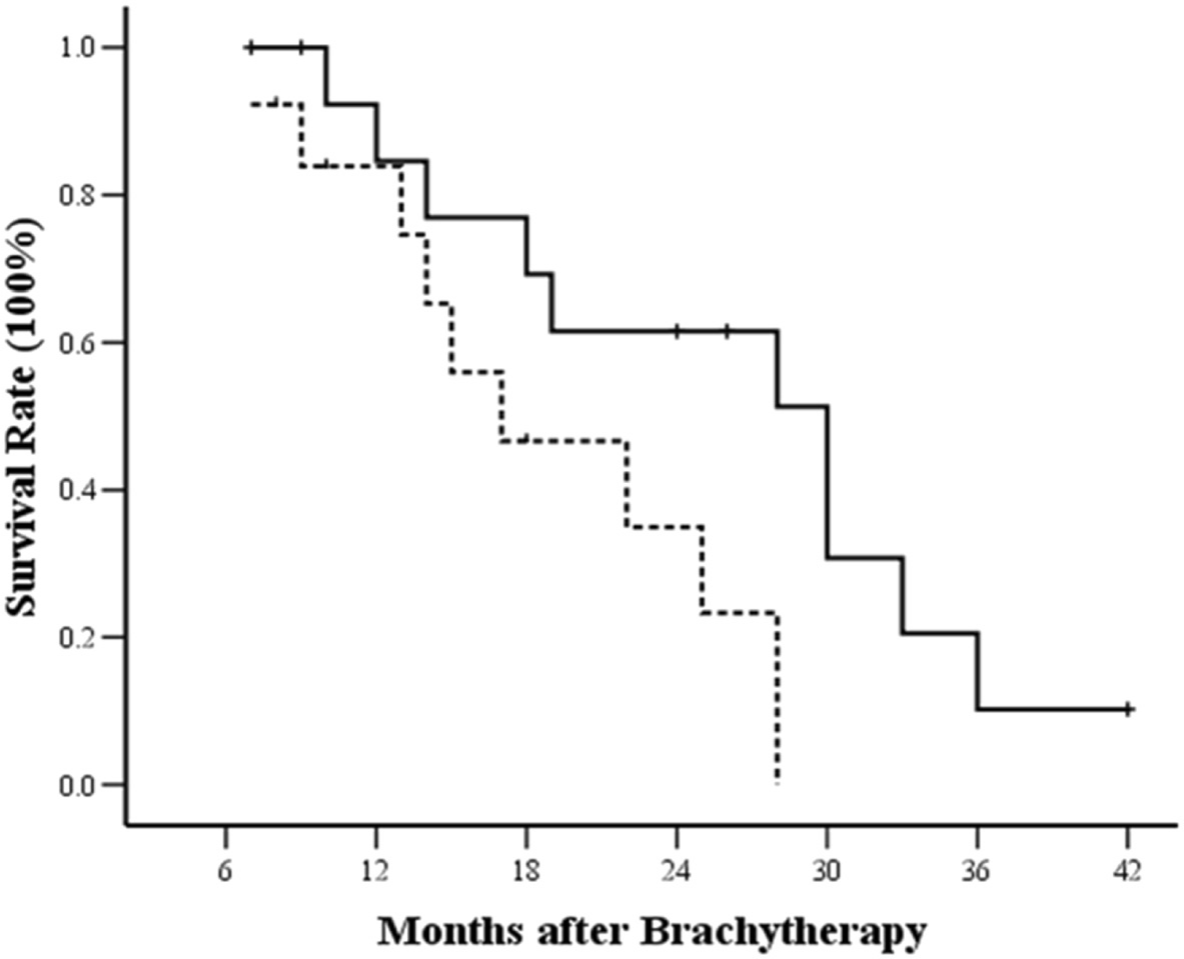
**Kaplan-Meier estimates of OS for patients divided into two subgroups according to tumor size (*P*** **= 0.026, log-rank test).** Continuous line: patients with tumor < 3 cm; dotted line, patients with tumor >3 cm.

At the end of follow-up, 20 patients had died. The median overall survival time was 22.0 ± 5.2 months for all patients, and the 1, 2 and 3-year survival rate was 89 ± 6%, 52 ± 10% and 11 ± 7%, respectively (Figure 2). Univariate Cox regression analysis showed that tumor size was a significant predictor for OS (*P* = 0.034). A cut-off point of 3 cm for the longest tumor diameter was found to be most useful in separating patients in terms of their survival, with patients having a tumor size less than 3 cm recurrent tumor demonstrating better survival (*P* = 0.026, Figure 3). The median survival time was 30.0 ± 5.1 months in the tumor size <3 cm group, and it was 17 ± 5.0 months in the >3 cm group. Patients with tumor size <3 cm had relatively higher complete response rate (66.7%, 10/15) compared to the patients with tumor size >3 cm (30.8%, 4/13).

During the follow-up, disease progression was detected in 27 patients. The median progression-free survival (PFS) after CT-guided brachytherapy was 11.4 ± 4.6 months, the 1, 2 and 3-year PFS rate was 50 ± 9%, 11 ± 6% and 4 ± 4%, respectively. Metastasis to other region was the most frequent recurrent pattern in the study. The first progression pattern after brachytherapy was extensive lymph node metastasis (9 cases), single regional lymph node recurrence (3 cases), heamatogenous metastasis (8 cases), ascites (3 cases), remnant stomach recurrence (1 case), multiple recurrence (3 cases), and progression in the initial recurrent region (2 cases). The median time to metastases to other regions was 13.5 ± 5.5 months in the tumor size <3 cm group, and 9.0 ± 3.5 months in the >3 cm group.

### Treatment parameters and complications

A total of 1145 seeds were implanted. The median PTV was 35.49 cm^3^ (ranging from 20.15 to 113.04 cm^3^). 15-75 (median 40) of 125I seeds were implanted into each patient to achieve MPD 100-160 Gy (median 120 Gy). 2-10 (median five) needle punctures were performed to distribute seeds into the tumors. Median day 0 dosimetry was as follows: V100 95.8% (90.2-120.5%) and D90 105.2% (98.0-124.6%) of prescription dose. The treatment parameters are shown in Table 2. In addition, the relationship of the dose-volume parameters to tumor response was reviewed. The value of PTV was significantly related to response of the target tumor. The complete response of target tumor was 25.0% (3/12) in patients with PTV ≥ 45 cm^3^, compared with 75.0% (12/16) in patients with PTV < 45 cm^3^ (*P* = 0.020). The V100 higher than 95% was associated with achieving complete tumor eradication, but the difference was not statistically significant (*P* = 0.056). Two month after seed implantation, 11 (73.3%) of 15 patients with V100 ≥ 95% achieved complete response in the treated region, compared with 4 (30.8%) of 13 patients with V100 < 95%. The relationship between tumor response and D90 was no significant at any cut-point analyzed.

**Table 2:** Treatment parameters

**Parameter**	**Median**	**Range**
GTV (cm^3^)	14.58	3.23-58.65
PTV (cm^3^)	35.49	20.15-113.04
MPD (Gy)	120	100-160
D90 (% of prescription dose)	105.2	98.0-124.6
V100 (% of PTV)	95.8	90.2-120.5
No. of seed	40	15-75
Puncture tracks	5	2-10
Activity of seed (mCi)	0.7	0.5-0.8
Total activity (mCi)	28.0	10.5-52.5

GTV: Gross Tumor Volume;PTV: Planning Target Volume; MPD:matched peripheral dose; PD prescription dose.

**Figure 4:**
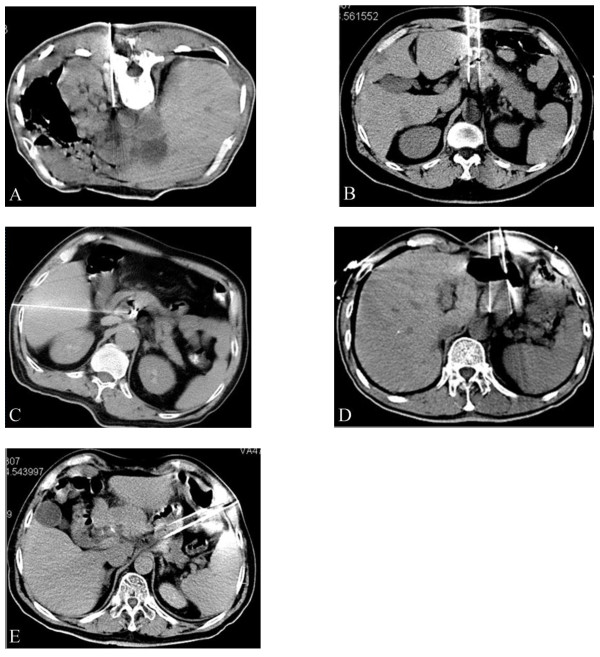
**Iodine-125 seeds were implanted through percutaneous puncture under CT-guidance: a**). Through the paraspinal space; **b**). Through the perirenal space between stomach and intestinal tract; **c**). By penetrating the liver; **d**). By traversing the stomach. **e**). By traversing the colon

Percutaneous puncture was carried out via the following methods: 1). Through the paraspinal space (in 10 cases); 2). Through the perirenal space or gaps between the stomach and intestinal tract (in 9 cases); 3. Penetrating the liver (in 4 cases); 4). Traversing the stomach (in 8 cases); 5). Traversing the colon (in 5 cases) (Figure 4). All the approaches were shown to be safe without serious complications such as haemorrhage, bile fistula, pancreatic fistula, and peritonitis. No patients died of perioperative complications. The mean hospital staying after treatment was 3.2 days (median 3 ± 0.5 days). During the follow-up, 14 (1.22%) of 1145 seeds had migrated in 3 (10.7%) of 28 patients. 6 (0.52%) seeds had migrated to the peritoneal cavity in one patient and 8 (0.70%) to liver in 3 patients. No adverse effects were observed in the 3 patients with seed migration. The common side-effects of radiotherapy such as gastrointestinal reactions and bone marrow depression were not observed before commencing chemotherapy. There was also no gastrointestinal bleeding or perforatin in the study.

4 patients received a second operation two months after the first seeds implantation (2 patients within the first brachytherapy PTV and 2 patients in para-aortic lymph node recurrence). The mean PTV of the second brachytherapy was 12.0 cm^3^ (5.5-20.8 cm^3^), and the average number of seeds implanted was 22 (ranging from 8 to 35). 24 patients received palliative chemotherapy based on Xeloda (n = 11) or S-1 (n = 13) after brachytherapy. The median duration of chemotherapy was 4 months (ranging from 2 to 10 months).

## Discussion

Local or regional recurrence in the tumor bed, the anastomosis, or regional lymph nodes occurs in 40- 65% of patients after gastric resection with curative intent [19,20]. Intraluminal local recurrence is rare but curable in 50% of cases without distant metastases. However, extraluminal locoregional recurrence comprises the major portion of recurrence and cannot be removed in most patients [21]. During the four years of recruitment for the trial, 28 patients diagnosed with limited extraluminal locoregional recurrent gastric cancer received CT-guided seed implantation in our hospital. Most of the recurrent tumors were located in or near the primary tumor bed, such as the bursa omentalis, the head of pancreas, the tail of pancreas, and the retroperitoneal lymph node. In this study, we demonstrated the clinical effectiveness of CT-guided brachytherapy as a salvage therapy for extraluminal locoregional recurrent gastric cancer.

Permanent implantation of radioactive seeds has emerged as a microinvasive local treatment modality, which has been successfully applied to many solid tumors such as prostate cancer, pancreatic cancer, non-small-cell lung cancer and metastatic tumors [13,16,22-24]. Percutaneous image-guided seeds implantation that can be performed without surgery or general anesthesia has attracted attention because of the advantages it offers in increasing the dose of radiation administered to tumors, without damaging neighboring organs [25]. With this technique, highly effective radiation doses are applied as a single fraction, ensuring protracted cell killing over a period of several weeks or even months. Compared to other interventional procedures, the advantages include: interference-free and accurately predictable energy distribution, treatable size of a target lesion, and lower rate of acute adverse effects by maintaining tissue continuity [14,16]. Prior and during the seeds implantation procedure, we carefully designed and chosed the safest paths for needle insertion. We used the natural gaps between organs as much as possible, followed by penetrating the liver and transversing the stomach or colon when. The potential complications of the treatment, including haemorrhage, bile fistula, pancreatic fistula, and peritonitis did not occurred in the study. Although Shah AP et al. [26] reported that bowel puncture did not correlate with the occurrence of acute or late toxicity, we rarely inserted the implant needles through the bowel. In our study, all the operations for radioactive seeds implantation were performed successfully under CT-guidance. No serious complications during the course of treatment.

Surgery and external beam radiotherapy (EBRT) are the main local treatment modalities that have been examined in the past studies. Due to the small number of cases suitable for local treatment, as well as the nature of the available treatments, it is challenging to design and complete a randomized prospective study on the local treatment of locoregional recurrent gastric cancer.

Although surgery is often recommended for the local recurrence of gastric cancer, the percentage of patients with locoregional recurrent gastric cancer in whom surgery can be attempted is low [27-29]. In the group studied by Yoo et al. [6], the proportion of patients treated with curative surgery was only at 3.7%. Carboni et al. [30] assessed 38 patients with solitary locoregional recurrent gastric cancer for macroscopical resection. The results showed that only 5 patients with intraluminal recurrence and 1 patient with extraluminal recurrence were resected with curative intent. However, postoperative complications occurred in two patients, and one patient died 35 days after surgery. Carboni et al. suggested that surgery plays a very limited role in the treatment of isolated locoregional recurrence of gastric cancer. Nunobe et al. [8] recently reported another retrospective study, in which the inclusion criteria were similar to ours. They reported that the 1, 3, and 5-year survival rates were 73.0%, 36.7%, and 9.8%, respectively, in 36 patients with solitary locoregional recurrent gastric cancer treated by surgery combined with chemotherapy. 36.1% of patients developed postoperative complications, and the average hospital stay was longer than 30 days (31.3 ± 5.3 days). Compared to our study, the proportion of patients with long-term survival greater than 3 years is higher in the Nunobe study (11% versus 36.7%). But the median survival time is similar (22.0 months versus 23.0 months). However, the percentage of patient suitable for surgery was not provided in the report, compared to the typical locoregional recurrence of gastric cancer.

Several clinical trials have confirmed that adjuvant chemoradiotherapy can prolong the survival of patients at high risk for recurrence of gastric cancer [3,31,32]. But, few published studies have assessed the efficacy of external beam radiotherapy (EBRT) to recurrent gastric cancer. Recently, Sun et al. [10] retrospectively reported that external beam radiotherapy (EBRT) with delivery of 50 Gy could prolong the survival of patients with abdominal lymph node (LN) metastases from gastric cancer. The results showed that the ORR was 83.8% (CR 29.7%, PR 54.1%), and median survival time was 11.4 months in the radiation group. Stereotactic body radiotherapy (SBRT) is one of the most advanced radiotherapy technologies available, and can deliver high, ablative doses of radiation in a limited number of fractions. Kim et al [11] reported that SBRT can produce significant local control in patients with isolated para-aortic lymph node (PALN) recurrence of gastric cancer. Since the patients included in our study were strictly selected with limited extraluminal recurrence, we consider that SBRT may also be a treatment option for most these patients if the technology is available. Another method that allows the delivery a high dose to tumor beds with minimal exposure of surrounding tissues is intra-operative radiotherapy (IORT). In a retrospective study reported by Miller et al. [33], 50 patients with locally advanced primary or recurrent gastric or esophageal adenocarcinomas received IORT given as a single fraction of electron beam radiotherapy (10-25 Gy), foliowing maximal tumor resection. The results showed that distant metastatic failure was 79%, local failure was 10%, and regional failure was 15%, and the 1, 2 and 3-year survival rate was 70%, 40%, and 27%, respectively. In summary, there is little published data available on radiotherapy for locoregional recurrent gastric cancer, and there are no prospective controlled studies that have compared radiotherapy to chemotherapy alone. Currently, it is still difficult to assess the role of radiotherapy in improving overall survival of patients with recurrent gastric cancer.

In our study, the tumor size was a significant factor associated with the overall survival of the patients with locoregional recurrent gastric caner treated with Iodine-125 seed brachytherapy. We found that a cut-off point of 3 cm of the largest tumor diameter was the best way to separate patients in terms of their survival. As we know, the local failure of radiotherapy is in part due to tumor hypoxia and cold spots in tumor subvolumes [34,35]. With an increase in tumor size, the hypoxic fraction usually increases, and a concurrent increase in radiation cold spot is also likely [36]. Since locoregional recurrent tumors of gastric cancer are often deep-lying and surrounded by vital structures, only a few needle paths could be explored for seed implantation in the study. Therefore, it may be challenging to ensure satisfactory seed distribution of within the tumor, especially when the tumor size is larger than 3 cm in diameter. As a result, cold spots may occur due to the unsatisfactory distribution of seeds. We reviewed the relationship of dose-volume parameters to tumor response, and verified a large PTV and a low V100 were associated with a high rate of tumor residual. Our results showed that the larger tumor size not only related to a higher local tumor residual rate, but also a shorter time to others metastases. In patients with tumor > 3 cm in size, tumor spreading to other regions occurred earlier than in patients with smaller recurrent tumors (the median time to other metastases was 13.5 months *vs* 9.0 months).This finding may be explained in two ways. First, a larger tumor size usually means that the tumor is late-stage, and therefore more likely to metastases. On the other hand, most of the patients with larger tumor had residual tumor after seed implantation, and the failure to completely eradicate the tumor might lead to subsequent shedding of tumor cells and a late wave of metastases [37].

A limitation of our study that cannot be ignored is the lack of a control group with similarly localized tumor recurrence, which was administered modern systemic chemotherapy. As our study population was too small for a randomized trial, it is only possible to compare this study’s results with those from the chemotherapy studies. Recently, several pivotal studies have demonstrated the effectiveness of S-1 based chemotherapy as a first-line treatment for the advanced or recurrent gastric cancer. Koizumi W et al. [38] reported a response rate of 54% (CR 1%, PR 53%), and median survival time of 13 months in patients with advanced gastric cancer who received S-1 plus cisplatin. Another phase III study showed a partial response rate of 41.5% (no patients achieved CR), and a median survival time of 12.8 months in patients treated with irinotecan plus S-1 as first-line treatment [39]. We also should mention that in the study reported by Shitara K [40], patients with recurrent gastric cancer after adjuvant chemotherapy were included, The response rate was 19.4%, and median survival time was 12.2 months treated with S-1 plus cisplatin. Although most of patients (24/28) included in our study received adjuvant chemotherapy after surgery, we still achieved a response rate of 78.6% (CR 50.0%, PR 28.6%), and the 1-year local control rate in PTV was 85.7% (24/28). These results may demonstrate the local control power of brachytherapy using ^125^I seeds implantation.

Although there was no control group in the present study treated solely with modern systemic chemotherapy, we think that CT-guided brachytherapy is effective in improving local control of the locoregional recurrent gastric cancer in selected patients. A randomized controlled trial with a larger sample size is needed to further verify these findings,

## Competing interests

The authors declare that they have no competing interests.

## Authors’ contributions

LRS carried out data acquisition, performed the statistical analysis, drafted the manuscript and participated in the sequence alignment. CPW conceived of the study, participated in the design of the study. JW, WJZ, MJ, HYZ, YQH and ZL participated in the sequence alignment. WJZ carried out data acquisition. HLP, JFJ and JTJ participated in its design and coordination and helped to draft the manuscript. All authors read and approved the final manuscript.
